# Effects of Chronic and Acute Ozone Exposure on Lipid Peroxidation and Antioxidant Capacity in Healthy Young Adults

**DOI:** 10.1289/ehp.10294

**Published:** 2007-09-11

**Authors:** Connie Chen, Mehrdad Arjomandi, John Balmes, Ira Tager, Nina Holland

**Affiliations:** 1 Division of Environmental Health Sciences, School of Public Health, University of California, Berkeley, California, USA; 2 Lung Biology Center, Department of Medicine, University of California, San Francisco, California, USA; 3 Division of Epidemiology, School of Public Health, University of California, Berkeley, California, USA

**Keywords:** antioxidant capacity, FRAP, isoprostane, lifetime exposure, lipid peroxidation, plasma, oxidative injury, ozone

## Abstract

**Background:**

There is growing evidence for the role of oxidative damage in chronic diseases. Although ozone (O_3_) is an oxidant pollutant to which many people are exposed, few studies have examined whether O_3_ induces oxidative stress in humans.

**Objectives:**

This study was designed to assess the effect of short-and long-term O_3_ exposures on biomarkers of oxidative stress in healthy individuals.

**Methods:**

Biomarkers of lipid peroxidation, 8-isoprostane (8-iso-PGF), and antioxidant capacity ferric reducing ability of plasma (FRAP) were analyzed in two groups of healthy college students with broad ranges of ambient O_3_ exposure during their lifetimes and previous summer recess either in Los Angeles (LA, *n* = 59) or the San Francisco Bay Area (SF, *n* = 61).

**Results:**

Estimated 2-week, 1-month, and lifetime O_3_ exposures were significantly correlated with elevated 8-iso-PGF. Elevated summertime exposures resulted in the LA group having higher levels of 8-iso-PGF than the SF group (*p* = 0.02). Within each location, males and females had similar 8-iso-PGF. No regional difference in FRAP was observed, with significantly higher FRAP in males in both groups (SF: *p* = 0.002; LA: *p* = 0.004). An exposure chamber substudy (*n* = 15) also showed a significant increase in 8-iso-PGF as well as an inhibition of FRAP immediately after a 4-hr exposure to 200 ppb O_3,_ with near normalization by 18 hr in both biomarkers.

**Conclusions:**

Long-term exposure to O_3_ is associated with elevated 8-iso-PGF, which suggests that 8-iso-PGF is a good biomarker of oxidative damage related to air pollution.

Oxidant generation is part of the normal metabolism of many cell types and is critical for homeostasis. Humans have evolved an antioxidant system to protect against oxidative stress—a state of imbalance between oxidant production and antioxidant defenses. Inhaled toxicants such as air pollutants and tobacco smoke can increase oxidative stress (Long et al. 2001; Morrow et al. 1995; Sorensen et al. 2003) through direct generation of reactive oxygen species (ROS) and through activation of inflammatory leukocytes. Inflammatory cells respond with the “respiratory burst” that involves the uptake of oxygen and release of ROS into the cellular surroundings. Inhaled air pollutants such as ozone interact directly with biomolecules in the respiratory tract lining fluid (RTLF), including unsaturated lipids, nucleic acids, and proteins (Mustafa 1990), damage that is reflected in peripheral blood.

Lipid peroxidation occurs when lipids are attacked by free radical species and hydrogen atoms are extracted from the methylene carbon side chain, initiating a cascade of free ROS that can cause oxidative damage to cell structures (Mudway and Kelly 2000; Pryor 1994). To terminate these events, radicals need to react with each other to create a nonradical species or be quenched by an antioxidant (Halliwell and Chirico 1993). This secondary inflammatory cell response to the initial oxidative damage results in F_2_-isoprostanes formation, which may contribute to lung injury.

F_2_-isoprostanes are a recently discovered series of bioactive prostaglandin F_2_-like compounds produced independently of the cyclooxygenase enzymes via arachadonic acid (AA) peroxidation (Morrow and Roberts 2002). A substantial portion of isoprostanes undergoes β-oxidation in tissues before their release into plasma. Of these, mainly 8-iso-prostaglandins-F_2α_(8-iso-PGF) are formed continuously under normal physiological conditions and can be found at elevated levels as a result of environmental exposures, including O_3_ and cigarette smoke (Chiabrando et al. 1999; Montuschi et al. 2000; Obata et al. 2000). Chamber studies have shown increased levels of 8-iso-PGF after 1 hr (Devlin et al. 1996; Hazbun et al. 1993) and 4 hr of exposure to O_3_ in healthy subjects (Montuschi et al. 2002). A recent study found increased plasma levels of 8-iso-PGF in both smokers and nonsmokers after multiday exposure to tobacco smoke (Ahmadzadehfar et al. 2006), an observation that suggests that chronic exposure may increase oxidative stress.

Clinical studies have shown that in addition to the peroxidation of AA, 8-iso-PGF also reflects the redox status of the microenvironment (Lawson et al. 1999). 8-iso-PGF and other indicators of redox status have been used as markers of asthma severity (Nadeem et al. 2003) and chronic obstructive pulmonary disease (COPD) (Nadeem et al. 2005; Rahman et al. 1996). Redox status can serve as an important marker of an individual’s response to oxidative stress and is cost-effective to analyze in large epidemiologic studies.

Overall antioxidant capacity is a potential modifying factor of an individual’s response to oxidative stress. One such marker is the ferric reducing ability of plasma (FRAP) assay (Benzie and Strain 1996) in which a ferric-to-ferrous ion reduction at low pH causes a colored ferrous–tripyridyltriazine (TPTZ) complex to form. Absorbance changes are linear over a wide concentration range in antioxidant mixtures, including plasma. The FRAP assay has been used in studies on effects of pesticide exposure (Ranjbar et al. 2002) and vitamin supplementation on cardiovascular health (Bub et al. 2002).

Chronic airway diseases [e.g., asthma and COPD (Rahman et al. 2006)] and systemic diseases [e.g., atherosclerosis and diabetes mellitus (Kaneto et al. 2007; Papaharalambus and Griendling 2007)] are associated with increased oxidative stress and lipid peroxidation.

In the present study we explore the association between O_3_ exposure and markers of lipid peroxidation and antioxidant capacity in healthy students from the University of California, Berkeley (UCB), who experienced different seasonal and geographic levels of O_3_ over their lifetimes and during recent summer vacation in either greater Los Angeles (LA) or the San Francisco Bay Area (SF), or were acutely exposed to O_3_ in a controlled environment. This is an extension of a larger project examining the effect of lifetime O_3_ exposure on lung function in young adults (Tager et al. 2005).

## Materials and Methods

### Study participants and sample collection

The overall design of the study, presented in detail by Tager et al. (2005), included 255 first-year undergraduates at UCB, who were recruited for enrollment over a 2-year period. In this biomarker study, we used a subcohort of participants (*n* = 120) enrolled between February and the first week of June 2002, each of whom provided blood samples (Table 1). Eligibility was based on the following: *a*) lifelong residence in either LA or SF, *b*) lifetime never smoker, and *c*) no history of chronic respiratory disease. The majority of participants were Asian (50%) or Caucasian (35%). Hispanics (*n* = 10), African Americans (*n* = 1), and subjects with other ethnic backgrounds (*n* = 7) were pooled together as “Others” for statistical analysis. Lifetime residents of SF composed 51% of the subjects; 56.6% of all subjects were females. By design, ages ranged from 18 to 22 years. During the first 8–12 days of each subject’s return to UCB from summertime residences in LA or SF (August–early September 2002), a trained phlebotomist collected small samples of peripheral blood. Plasma was separated from red blood cells and stored in aliquots at −80°C until use. Spirometry was performed as previously described (Tager et al. 2005) and included lung function measures of forced expiratory volume in 1 sec (FEV_1_), forced expiratory flow rate between 25 and 75% (FEF_25–75_), forced expiratory flow rate at 75% (FEF_75_), and forced vital capacity (FVC). We also obtained a complete history of summertime (June–August) residences.

### Exposure assessment

The details for the assessment of lifetime exposure to O_3_ have been described previously (Kunzli et al. 1997; Tager et al. 2005). We reconstructed lifetime residential history with a standardized questionnaire; air pollutant [O_3_, particulate matter with an aerodynamic diameter of ≤10 μm (PM_10_), and nitrogen dioxide (NO_2_)] concentrations were assigned for each month of life to each residential location. Air quality data were acquired from the California Air Resources Board (ARB, compact disc no. PTSD-02-017-CD), the Aerometric Information Retrieval System, and from special requests to ARB. We interpolated monthly mean measures of O_3_ spatially from air quality monitoring stations to the residence locations with inverse distance weighting and a maximum of three monitoring stations for each interpolation (maximum interpolation radius of 50 km). Lifelong residents of LA had significantly higher estimated lifetime O_3_ exposure than SF residents (Table 1). Based on the same interpolation methods, we calculated estimates of short-term exposure to O_3_ based on the moving averages of 8-hr maximum O_3_ concentrations 1–30 days prior to the day of blood collection. Although both 1- and 8-hr maximum O_3_ average levels in LA (0.12 and 0.10 ppm, respectively) during summer were almost double those of SF (0.07 and 0.05 ppm, respectively), individual exposures from up to 1 month before blood collection from subjects in this study overlapped significantly (Table 1).

### Controlled O_3_ exposure substudy

The design of this substudy has been described previously (Chen et al. 2006). Briefly, we collected peripheral blood from 15 volunteers from the main cohort before and 18 hr after 4 hr of exposure to 200 ppb O_3_ in a chamber with intermittent exercise (30 min of each hour). Four subjects also donated blood immediately after exposure. We conducted chamber studies during the spring when ambient exposures are relatively low and when all study participants had been in SF for several months. Nine of the 15 subjects grew up in SF.

The Committee of the Protection of Human Subjects, UCB, and the Committee on Human Research, University of California, San Francisco, approved all protocols for this study, and we obtained written informed consent from all subjects.

### 8-Iso-PGF and FRAP assays validation and quality assurance

We conducted several pilot experiments prior to the study. The precision of both the 8-iso-PGF and FRAP assays, expressed as the coefficient of variation (CV% = (SD ÷ mean) ×100), was determined from triplicate assays of repeated samples from five laboratory controls with characteristics similar to those of the main study population. Separate plates were analyzed, and the CV% for the same control sample across several experiments fell below 10% for 8-iso-PGF and below 7% for FRAP. We performed additional quality assurance/ quality control (QA/QC) studies to determine if any artifactual formation of 8-iso-PGF levels in plasma could result from sample processing and storage. We compared four conditions for biological processing over a 7-day period: *a*) storage at −80°C after separation of plasma from red blood cells immediately after blood draw; *b*) storage at −20°C immediately; *c*) blood samples left at room temperature 5 hr, then at −80°C for the remainder of 7 days; and *d*) storage at −4°C for 5 hr, then at −80°C, mimicking the biological sample processing condition for this study. All conditions showed similar results with good reproducibility. Results of storage over the 2-week pilot study were comparable with those stored for several months and those reported in previously published studies. All samples, internal positive controls, and randomly distributed blanks were run in triplicate (variability < 3%, 8-iso-PGF, and < 5%, FRAP).

### 8-Iso-PGF assay

Lipid peroxidation was measured by a competitive enzyme-linked immunosorbent assay (ELISA) for 8-iso-PGF with a commercial kit (Cayman Chemical, Ann Arbor, MI). To prevent cross-reactivity, we purified plasma samples by affinity sorbent chromatography columns in accordance with the manufacturer’s protocol. The assay is based on the competition between 8-iso-PGF and 8-isoprostane–acetylcholinestase (AChE) conjugate for a limited number of binding sites in each ELISA plate well. The concentration of 8-iso-PGF is inversely proportional to the number of binding sites available, whereas AChE is held constant. Samples were applied to the column followed by several washes. The last wash that contained purified 8-iso-PGF in the eluate was dried by vacuum centrifugation. We reconstituted the resulting pellet in phosphate buffer (Cayman Chemical, Ann Arbor, MI) and transferred to the ELISA plate. The absorbance of the colorimetric enzymatic reaction was read at 405 nm using the SpectraMax Plus microplate reader (MDS Analytical Technologies, Sunnyvale, CA) and compared with an 8-iso-PGF standard curve to calculate concentration.

### FRAP assay

Antioxidant capacity was measured with the FRAP assay (Benzie and Strain 1996). Nonhemolyzed plasma samples were selected and analyzed in triplicate. Color change in the biological sample, measured at 593 nm wavelength 4 min after adding the FRAP reagent (acetate buffer, TPTZ, FeCl_3_ · H_2_O), is directly proportional to the combined reducing power of the antioxidants in the reaction mixture. Samples were run against internal antioxidant standards (α-tocopherol, Fe(II), uric acid, L-ascorbic acid). The analysis was performed in a 96-well plate with the SpectraMax Plus microplate reader.

### Statistical analysis

We performed statistical analysis with SAS 9.1 software (SAS Institute Inc., Cary, NC). FRAP levels were distributed normally; 8-iso-PGF levels were log-transformed to normalize the distribution. Student *t*-test was used to determine log 8-iso-PGF and FRAP differences by sex and geographic location. The nonparametric Wilcoxon signed-rank tests for 8-iso-PGF levels yielded the same results as the *t*-tests (results not shown). We used analysis of variance (ANOVA) to determine differences between the three ethnicity groups (coded as dummy variables: 0 = Caucasian, 1 = Asian American, 2 = Other). Bivariate analysis and correlations between log 8-iso-PGF, body mass index (BMI), weight, FRAP, and daily exposure to O_3_ up to 30 days before blood collection were determined by Pearson correlation coefficients. Multivariable analyses were conducted after examination of potential effect modifiers. Sex, ethnicity, BMI, FRAP levels, and O_3_ exposure estimates were included if they were statistically significant based on type III sum of squares. We ran models that included weight rather than BMI, but the results did not change significantly. However, because weight and BMI are collinear, we chose to use BMI, as it takes into account both height and weight. We also explored the effect of these biomarkers on lung function multivariable regression based on models that have been optimized previously for this cohort (Tager et al. 2005). The effect of age was not adjusted for in these models because of the narrow age range of subjects in the study.

## Results

**Figure 1:**
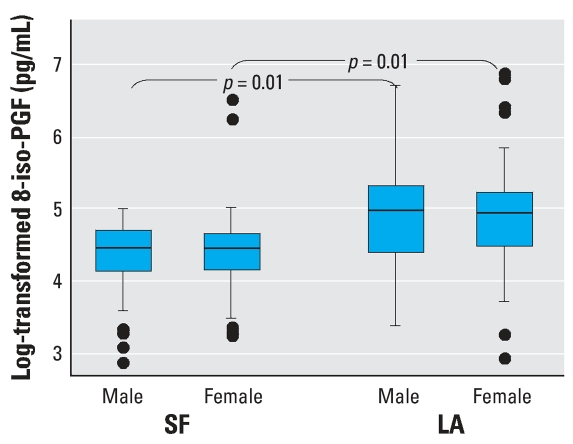
Boxplot distribution of levels of log-transformed 8-iso-PGF stratified by sex in two groups of students who spent their summer break in two geographic locations, SF (*n* = 61) and LA (*n* = 51). Overall, subjects from LA had significantly higher levels of 8-iso-PGF than those from SF (*p* = 0.02). The box represents the interquartile range; the horizontal line inside the box represents the median; and the vertical lines represent the minimum and maximum values.

**Figure 2:**
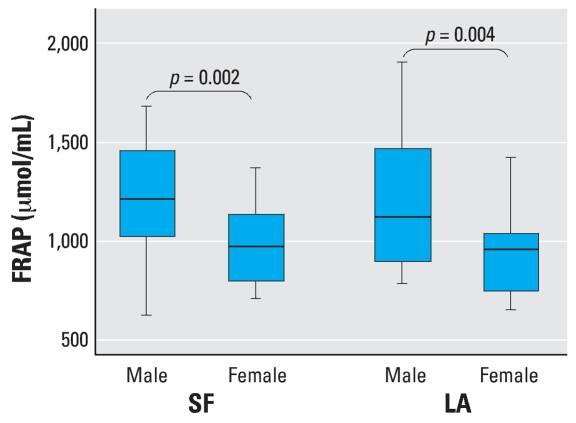
Boxplot distributions of levels of FRAP by sex in two groups of students who spent their summer break in two geographic locations, LA and SF. Overall, men (*n* = 52) had significantly higher FRAP levels than women (*n* = 68, *p* = 0.002). The box represents the interquartile range; the horizontal line inside the box represents the median; and the vertical lines represent the minimum and maximum values.

The distributions of the two biomarkers of oxidative stress, 8-iso-PGF and FRAP, in the study population are presented in Figures 1 and 2. Levels of 8-iso-PGF had wide interindividual variability (range, 17.4–940.7 pg/mL). Subjects from LA who were exposed to twice the level of O_3_ during the summer compared with those spending their summer in SF had, on average, 2-fold higher 8-iso-PGF (Table 2, *p* = 0.02, unpaired *t*-test). Levels of 8-iso-PGF did not vary significantly by sex (*p* = 0.81). Comparisons of 8-iso-PGF levels between sexes by the two geographic regions, LA and SF were not significant (*p* = 0.78 and *p* = 0.42 for men and women, respectively). Although there was no significant difference in 8-iso-PGF levels between the two predominant ethnicities, Caucasian and Asians, there was a suggestion that the “Other” ethnic group had increased 8-iso-PGF levels (*p* = 0.07). However, because of the small numbers, no one subgroup of the heterogeneous “Other” group could be identified as responsible for the difference. No correlation was observed between individual 8-iso-PGF and weight (*r* = 0.10, *p* = 0.27) or BMI (*r* = 0.12, *p* = 0.19).

FRAP levels ranged from 637.2 to 1908.9 μmol/mL. In contrast to 8-iso-PGF, FRAP levels did vary by sex (Figure 2B, *p* = 0.002) but not by geographic region (Figure 1B, *p* = 0.52). However, when comparisons between sexes by the two geographic regions were carried out, males had higher levels in both regions (*p* = 0.009 and *p* = 0.003, respectively). FRAP was also significantly correlated with weight (*r* = 0.40, *p* = 0.0002) and marginally correlated with BMI (*r* = 0.19, *p* = 0.09). Ethnicity was not associated with antioxidant capacity (Table 2, *p* = 0.32). FRAP levels were not correlated with 8-iso-PGF (*r* = −0.08, *p* = 0.47).

We estimated individual O_3_ exposures for up to 30 days prior to blood collection based on each subject’s summer residence and date of return to SF. Level of 8-iso-PGF showed the strongest associations with 2-week [β = 0.035 (pg/mL)/8-hr ppb O_3_, *p* = 0.007] and 1-month [β = 0.031 (pg/mL)/8-hr ppb O_3_, *p* = 0.006] prior O_3_ exposure estimates, despite significant overlap between the two geographic categories. However, estimated lifetime exposure had the most precise relation with 8-iso-PGF [β = 0.025 (pg/mL)/ppb O_3_, *p* = 0.0007]. There is a 17.41-pg/mL (95% CI, 15.43–19.39 pg/mL) increase in 8-iso- PGF for the 17-ppb cumulative lifetime O_3_ exposure difference between LA and SF subjects. Level of FRAP, on the other hand, was not associated with recent O_3_ exposures {2-week [β = –7.93 (pg/mL)/8-hr ppb O_3_, *p* = 0.95] and 1-month [β = 1.69 (pg/mL)/8-hr ppb O_3_, *p* = 0.76] prior} or lifetime exposure [β = –2.21 (pg/mL)/ppb O_3_, *p* = 0.45]. Correlations between the three O_3_ exposure estimates are presented in Supplemental Material, Table 1 (http://www.ehponline.org/docs/2007/10294/suppl.pdf).

The relations between biomarkers of oxidative stress and separate O_3_ (2 weeks prior, 1 month prior, estimated lifetime) exposure estimates were explored further with multivariable regression to control for the following covariates: sex, ethnicity, BMI, and FRAP. O_3_ exposure estimates were the strongest predictors for level of 8-iso-PGF (Table 3). A final model that included all three exposure metrics further confirmed that each exposure period had an independent association with 8-iso-PGF levels. The covariates did not contribute significantly to any model and their exclusion did not change the magnitude of the associations [Supplemental Material, Table 2 (online at http://www.ehponline.org/docs/2007/10294/suppl.pdf)].

The contribution of the estimated lifetime O_3_ exposure, independent of the recent 2-week and 1-month O_3_ exposures, was determined by evaluation of the distribution of residuals from a separate regression model that used the two short-term exposures as independent variables and lifetime exposure as the outcome [Supplemental Material, Table 3 (http://www.ehponline.org/docs/2007/10294/suppl.pdf)]. The lifetime exposure residuals obtained from this model were used as the O_3_ exposure variable in a final multivariable model and confirmed an independent effect of cumulative lifetime exposure [β = 0.025 (pg/mL)/ppb O_3_, *p* = 0.004].

Increased FRAP in females was marginally associated with lower lung function, FEF_75_ [Supplemental Material, Figure 1A; β = −0.0001 (μmol/mL)/(mL/sec), *p* = 0.15], after the removal of an outlier. In males, however, FRAP levels paralleled FEF_75_, although it was not statistically significant [Supplemental Material, Figure 1B; β = −0.0001 (μmol/mL)/ (mL/sec), *p* = 0.67]. No relation between 8-iso- PGF and FEF_75_ was found in either sex [Supplemental Material, Figure 2 (http://www.ehponline.org/docs/2007/10294/suppl.pdf)].

**Figure 3:**
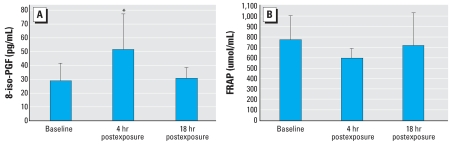
Distribution of (*A*) 8-iso-PGF and (*B*) FRAP after acute exposure to 200 ppb for 4 hr (*n* = 15). Data are presented as mean ± SD. *Mean value significantly different than other time points.

Biomarker levels from the acute exposure substudy are presented in Figure 3. The levels of 8-iso-PGF increased from 28.5 pg/mL at baseline to 51.1 pg/mL immediately after O_3_ exposure ended (*p* = 0.10) and by 18 hr had returned close to baseline (30.5 pg/mL, Table 4). There was a (22%) decrease in FRAP immediately after exposure (*p* = 0.17). Antioxidant capacity returned to near pre-exposure levels by 18 hr (723.5 vs. 771.4 μmol/mL, respectively). Individual FRAP values before and 18 hr after exposure were significantly correlated (*r* = 0.82, *p* < 0.0001); however, 8-iso-PGF measures were not (*r* = 0.48, *p* = 0.20). Correlations between levels at baseline and 4 hr after exposure, although relatively high, were not statistically significant for either FRAP (*r* = 0.77, *p* = 0.23) or 8-iso-PGF (*r* = 0.71, *p* = 0.23), possibly because of a relatively small number of subjects in the chamber study. Overall, average FRAP and 8-iso-PGF levels were lower in this controlled exposure substudy than in the larger cohort study. However, these same subjects were on the lower end of the isoprostane distribution for the entire study group. Additionally, the acute exposures were all conducted during the spring in SF when ambient pollution levels are low. Furthermore, 9 of the 15 subjects were lifetime SF residents with comparatively less O_3_ exposure than LA subjects.

## Discussion

Previously, we demonstrated that estimated lifetime exposure to ambient O_3_ in a cohort of young healthy adults was associated with reduced measures of lung function that reflect the physiology of the small airways, FEF_75_ and FEF_25–75_ (Tager et al. 2005). We also found that residence during summer seasons of elevated O_3_ in two geographic regions differentially affected cytogenetic damage in oral epithelia (Chen et al. 2006). Subjects who spent the summer in LA had significantly higher micronucleus frequencies than those who stayed in SF. Here, we observed a similar group effect of high oxidant region (summers in LA vs. SF) on elevated lipid peroxidation, as measured by 8-iso-PGF, in the same cohort. Addtionally, we determined if an individual’s exposure over varying periods of time affected his/her 8-iso-PGF levels. Multivariable regression demonstrated that estimated 2-week prior, 1-month prior, and lifetime O_3_ exposures of young healthy individuals were significantly and independently associated with increased 8-iso-PGF levels, while controlling for sex and ethnicity. In particular, these findings indicate that long-term exposure to elevated O_3_ can contribute to oxidant burden over periods longer than 1 month. Results from the controlled exposure substudy confirmed that oxidative stress increases after 4 hr of O_3_ exposure. Levels of 8-iso-PGF returned to near baseline by 18 hr without further exposure.

Although free 8-iso-PGF in humans has a relatively short half-life (~ 16 min), the time course observed in the participants of the acute study mirrored those reported in previous animal models of oxidant stress (Morrow et al. 1992). In the larger cohort study, we expected an individual’s most recent exposure (1- to 7-day time lag) prior to sample collection to correlate best with 8-iso-PGF. Contrary to this expectation, we observed significant effects for slightly longer recent O_3_ exposures (2 weeks and 1 month prior to blood collection) on 8-iso-PGF. Somewhat surprisingly, lifetime exposure also had a significant association with increased levels of this biomarker of lipid peroxidation, possibly because of greater precision for lifetime estimate than that for shorter lag times.

Even though we cannot be certain that the lifetime exposure associated with repeated oxidant injury has led to a chronic state of oxidative stress that persists over many years, the data do indicate chronic exposure of at least 2–4 weeks contributes to subacute oxidative stress. The observed oxidant effects on lipid peroxidation are consistent with those in other studies examining chronic oxidative stress. Ahmadzadehfar et al. (2006) reported that 8-iso-PGF levels increased during passive exposure of nonsmokers to cigarette smoke but tended to return to baseline by 6 hr post-exposure. After repeated exposure over 12 days, however, 8-iso-PGF levels in these nonsmokers rose to levels that approached those of active smokers. These results, together with ours, suggest that repeated exposures to oxidant pollutants can lead to sustained oxidative stress that, in turn, causes increased lipid peroxidation. The only other long-term study of the effect of air pollution on lipid peroxidation found a 30% decrease in the thiobarbituric acid reactive substances (TBARS) assay with extended stay in highly polluted areas of Mexico City (Medina-Navarro et al. 1997). The TBARS assay measures a different lipid peroxidation end point, malondialdehyde, and has been shown to be nonspecific and provide erroneously high estimates (Esterbauer 1996; Janero 1990).

O_3_ alone also may not be responsible for the associations observed. As we reported previously, in California the O_3_ season does not overlap with that of PM_2.5_ but does coincide with the seasonal rise of PM_10–2.5_ and NO_2_ (Tager et al. 2005), and these air pollutants are highly correlated in this study population [Supplemental Material, Table 4 (http://www.ehponline.org/docs/2007/10294/suppl.pdf)]. Additional multivariable analysis confirmed a significant effect of these co-pollutants; however, their inclusion in the models did not change the magnitude of the associations with O_3_. Furthermore, the validity of the association with ambient O_3_ exposure estimates in our study were strengthened by chamber substudy results, where O_3_-induced 8-iso-PGF was also increased after 4 hr of exposure compared with baseline. Here, the effect of O_3_, is clear and cannot be attributed to other pollutants. These results are consistent with those of other chamber studies that have observed an increase in lipid peroxidation after acute O_3_ exposure (Corradi et al. 2002; Devlin et al. 1996; Hazbun et al. 1993; Montuschi et al. 2002).

Our FRAP data showing that subjects living in LA during the summer had decreased antioxidant capacity compared with those who stayed in SF corroborate an earlier study by Medina-Navarro et al. (1997). Their study showed decreased antioxidant status, measured by serum superoxide dismutase, in subjects living in highly polluted areas compared with that in controls. Additionally, we observed a mild suppression of antioxidant levels, as measured by the FRAP assay, in the chamber substudy immediately after 4 hr of exposure to O_3_. Although no other studies have used the FRAP assay to assess the effect of air pollution on antioxidant capacity, β-carotene supplementation has been shown to increase FRAP levels (Bub et al. 2002), and exposure to pesticides caused levels to decrease (Ranjbar et al. 2002).

We observed broad interindividual variability in both biomarkers of oxidative stress, and explored the role of host factors such as sex, ethnicity, weight, and BMI. We found no significant associations between 8-iso-PGF levels and these covariates in either bivariate or multivariable regression analyses. In contrast, other studies of older adult subjects have reported that females have higher levels of 8-iso-PGF than males (Berr et al. 1998b; Block et al. 2002; Coudray et al. 1997). This effect of sex may be due to higher body mass and percentage fat in adult women, as BMI was associated significantly with lipid peroxidation in one study (Block et al. 2002). The women in our population of healthy young students, however, tended to have low BMI levels.

The men in our study had significantly higher antioxidant capacity than females, as measured by FRAP, that could have masked an overall effect of sex on 8-iso-PGF. The role of sex in antioxidant capacity was reported previously in a population of older adults (Berr et al. 1998a). We observed a moderate correlation between FRAP and BMI (*r* = 0.19, *p* = 0.09) and a significant correlation between FRAP and weight (*r* = 0.40, *p* < 0.0001). Furthermore, overall antioxidant levels were not significantly correlated with 8-iso-PGF levels. This suggests that an aggregate measure of antioxidant capacity such as FRAP may not be as informative to an individual’s lipid peroxidation status in response to O_3_ (Mudway and Kelly 2000). However, total antioxidant capacity have been used successfully to characterize disease status in asthma and COPD (Nadeem et al. 2003; Rahman et al. 1996).

Although O_3_ exposure has been demonstrated to affect lipid peroxidation in this study, another factor that may contribute to interindividual variability is self-reported ethnicity. The majority of subjects were either Caucasian or Asian, which reflects the demographics of the UCB student population. While we observed a marginal effect of race/ ethnicity on 8-iso-PGF (“Others,” *p* = 0.07), we acknowledge that self-reported race/ ethnicity is not a precise biological concept (Hanis et al. 1986; Hoggart et al. 2003; Parra et al. 2001; Pritchard et al. 2000). One study reported that African Americans have lower 8--iso-PGF levels than Caucasians (Block et al. 2002), whereas other studies have not observed any differences among ethnicities (Ke et al. 2003; Lopes et al. 2003). Some of the variability seen in this study may be explained by polymorphisms of genes involved in antioxidant defenses, many of which vary by race/ethnicity. For example, we found that both genotype frequencies and enzyme activity of manganese superoxide dismutase, a polymorphic enzyme involved in antioxidant defense, varied by ethnicity in this same cohort (Bastaki et al. 2006). The effects of such polymorphisms on lipid peroxidation are currently being explored.

The relations between 8-iso-PGF, FRAP, and previously established O_3_-induced cytogenetic damage (Chen et al. 2006) and lung function decrements (Tager et al. 2005) were also assessed. Lipid peroxidation was not associated with lung function [Supplemental Material, Figure 2 (online at http://www.ehponline.org/docs/2007/10294/suppl.pdf)] and only marginally associated with cytogenetic damage as assessed by the micronucleus frequency during the fall (*r* = 0.15, *p* = 0.09; Spearman correlation). FRAP was inversely associated with cytogenetic damage (*r* = –0.21, *p* = 0.06) and lung function [Supplemental Material, Figure 1 (online at http://www.ehponline.org/docs/2007/10294/suppl.pdf)].

The results of both the main chronic study and the chamber substudy presented here provide additional evidence that inhalation of O_3_ causes lipid peroxidation that can be detected in peripheral blood. 8-Iso-PGF appears to be a good biomarker of the oxidative damage related to inhaled O_3_ and high oxidant environments. We have demonstrated an effect of a single controlled O_3_ exposure and chronic ambient exposure on elevated 8-iso-PGF. Additional studies involving single, acute exposures that examine the 8-iso-PGF time course and repeated acute exposures in humans are warranted to elucidate the true effect of chronic exposure and accumulation of 8-iso-PGF over time. The implications of the results presented here are particularly significant because oxidative stress that results from exposure to ambient pollutants may contribute to lung cancer, asthma, and cardiopulmonary morbidity and mortality (Dominici et al. 2003; Kunzli et al. 2003; Pope et al. 2002; Samet et al. 2000).

## Figures and Tables

**Table 1:** Cohort characteristics.

Characteristic	LA	SF	Total
No. of subjects	59	61	120
Sex (% female)	56.9	57.3	56.6
Ethnicity (%)			
Caucasian	45.8	54.1	50
Asian	30.5	41.0	35.8
Other	23.7	4.9	14.5
Age (years)	19 (19–20)	19 (18–22)	19 (18–22)
Weight (kg)	62.3 (46.8–97.0)	62.5 (40.9–113.9)	62.4 (40.9–113.9)
BMI	22.4 (17.2–37.4)	21.8 (16.8–32.2)	22.2 (16.8–37.4)
Recent O_3_ exposures (8-hr moving averages, ppb)			
2 week	30.7 (14.3–43.1)	30.9 (13.5–47.9)	30.8 (13.5–47.9)
1 month	28.4 (5.0–41.8)	28.1 (14.1–43.1)	28.3 (5.0–43.1)
Lifetime exposure (estimated monthly average, ppb)			
O_3_	42.9 (28.5–65.3)	26.9 (17.6–33.5)	30.5 (17.6–65.3)
PM_10_ (before 1987)	92.0 (63.9–124.2)	52.6 (34.2–89.6)	68.1 (34.2–124.2)
PM_10_ (after 1987)	42.3 (25.7–67.9)	25.6 (17.8–28.6)	28.5 (17.3–67.9)
NO_2_	39.7 (8.3–49.9)	21.6 (11.4–29.6)	26.9 (8.3–49.9)

Data are presented as either percentage or median (range).

**Table 2:** Biomarkers of lipid peroxidation (8-iso-PGF) and antioxidant capacity (FRAP).

Parameter	8-Iso-PGF^a^,^b^	FRAP^a^,^c^
Geographic location		
SF (*n* = 59)	97.2 (17.4–674.7)*	1,059.4 (637.2–1686.0)
LA (*n* = 61)	195.3 (18.6–940.7)	1,002.5 (660.1–1908.9)
Sex		
Male (*n* = 52)	133.1 (17.4–807.2)	1,196.3 (637.2–1908.9)*
Female (*n* = 68)	154.9 (18.6–940.7)	970.8 (660.1–1429.5)
Ethnicity		
Caucasian (*n* = 42)	126.1 (18.6–940.7)	1,041.4 (637.2–1686.0)
Asian (*n* = 60)	169.4 (14.4–877.5)	1,065.5 (685.8–1908.9)
Other (*n* = 18)	154.1 (34.9–574.1)	884.1 (757.5–1686.0)

a Data are presented as median (range).

b Raw values presented in pg/mL; log–transformed concentrations were used for regression analyses.

c Values are μmol/mL.

* *p* < 0.05.

**Table 3:** Exposure models for predictors of 8-iso-PGF in healthy young adults.

	Model			
O_3_ measure	1	2	3	Full
2 weeks	0.035 (0.015)*			0.023 (0.010)*
1 month		0.031 (0.013)*		0.025 (0.009)*
Lifetime			0.024 (0.008)*	0.023 (0.006)*

Data are presented as coefficient (SE). Outcome of 8-iso-PGF was log-transformed to normalized distribution. Units for parameter estimates are log 8-iso-PGF/change in O3 measure (8-iso-PGF: pg/mL; O_3_ measure: 2 weeks, 1 month = ppb 8-hr maximum; lifetime exposure ppb-hr). Separate models were run for each exposure metric. The full models that include other covariates (sex, BMI, etc.) can be found in Supplemental Material (http://www.ehponline.org/docs/2007/10294/suppl.pdf).

* *p* < 0.05.

**Table 4:** 8-Iso-PGF and FRAP after acute exposure to 200 O_3_ ppb for 4 hr (*n* = 15).

	Preexposure	4 hr postexposure^a^	18 hr postexposure
8-iso-PGF (pg/mL)	28.5 ± 12.5	51.1 ± 25.1*	30.5 ± 7.4
FRAP (μmol/mL)	771.4 ± 234.7	600.3 ± 86.4	723.5 ± 205.8

Data are presented as mean ± SD.

a *n* = 4.

* *p* < 0.10, ANOVA (Tukey post hoc) compared with preexposure and 18 hr postexposure.
